# Supplementary material for: Clinical Metagenomics Is Increasingly Accurate and Affordable to Detect Enteric Bacterial Pathogens in Stool
Source: Microorganisms. 2022 Feb 15;10(2):441. doi: 10.3390/microorganisms10020441 (PMC8880012; doi:10.3390/microorganisms10020441)
Supplement: Supplementary file 1 [file microorganisms-10-00441-s001.zip › Supplementary Figure S1-S3.pdf]

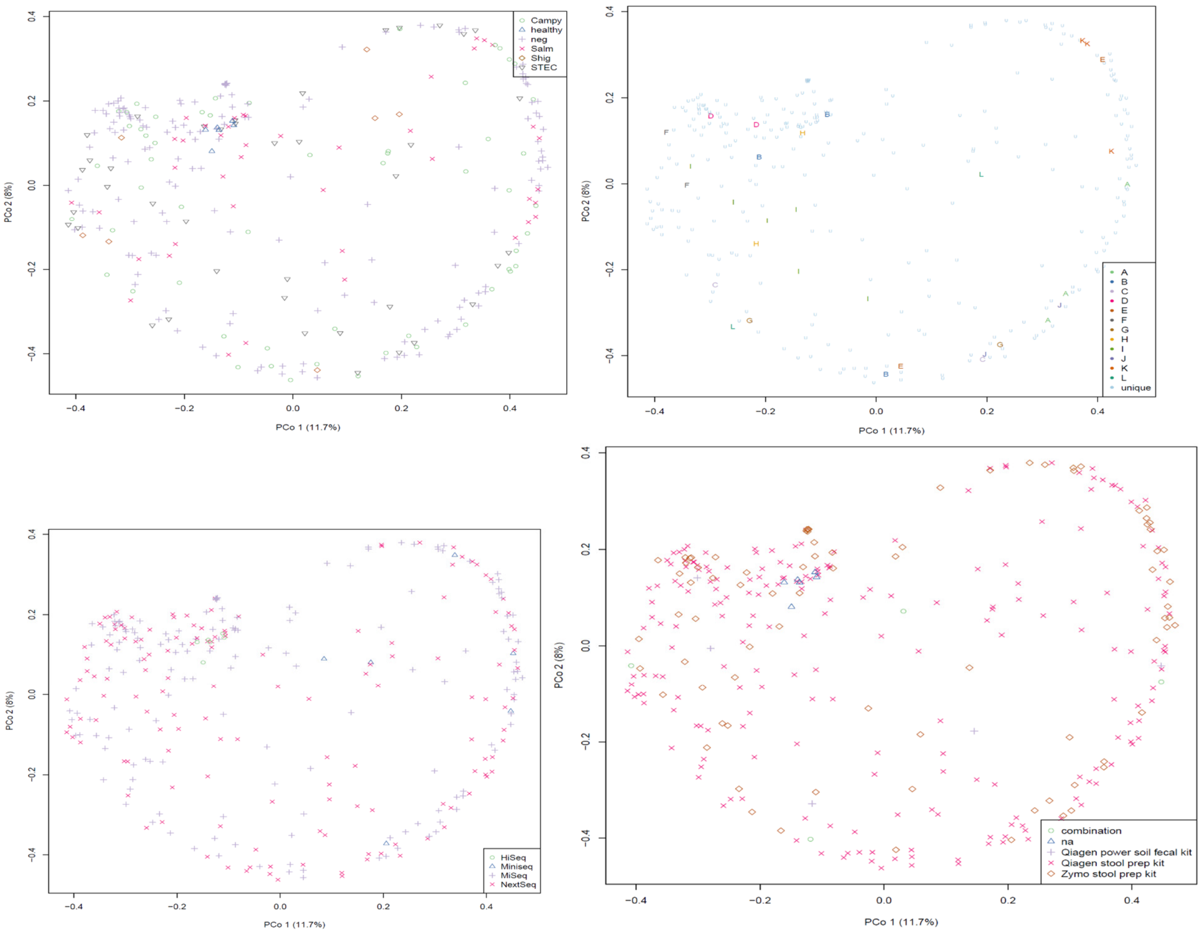

Figure S1. Principle coordinate analysis (PCoA) using the Bray Curtis Dissimilarity distance measure among trimmed and normalized metagenomes on groups within each variable, stringent filtering.

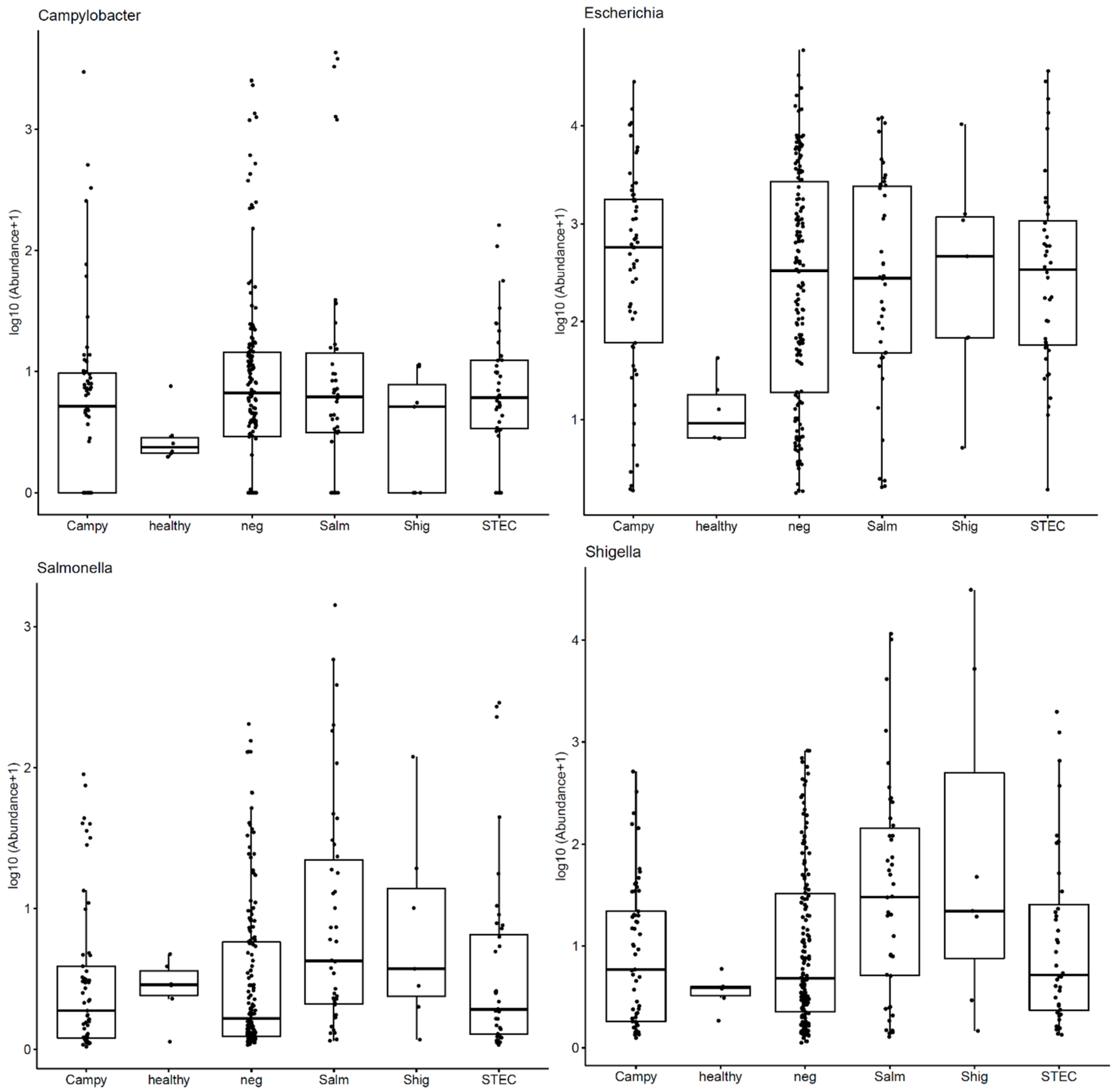

Figure S2. Abundance of reads classified as Campylobacter (A), Escherichia (B), Salmonella (C), and Shigella (D) as detected in stool metagenomes from Campylobacter culture-positive (Campy), Salmonella culture-positive (Salm), Shigella culture-positive (Shig), STEC culture-positive, culture-negative, and healthy specimens (healthy).

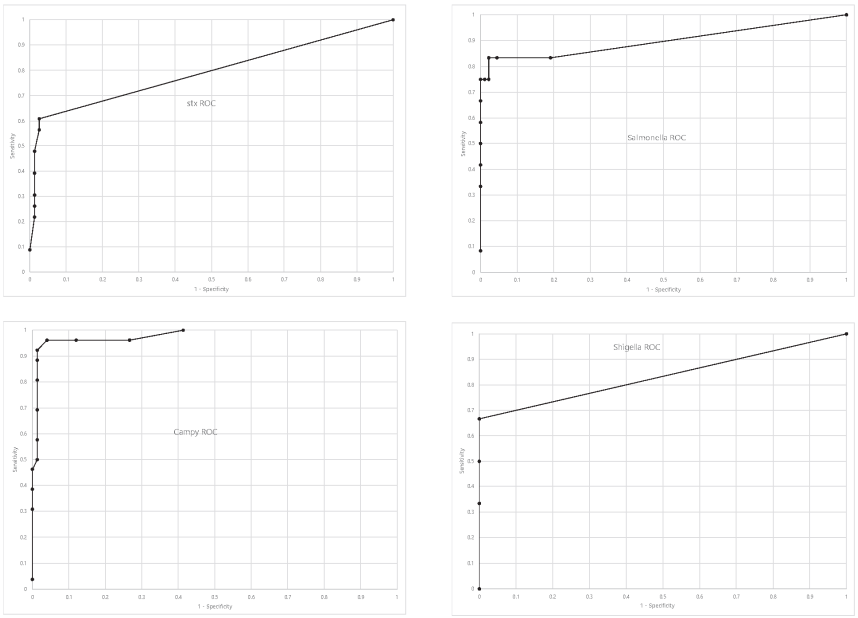

Figure S3. Receiver operator characteristic (ROC) curves used to measure the k-mer coverage threshold resulting in the highest sensitivity and specificity for the training set of 104 specimens. (A) Salmonella, (B) Campylobacter, (C) Shigella and (D) STEC.
